# Occlusion of left atrial appendage affects metabolomic profile: focus on glycolysis, tricarboxylic acid and urea metabolism

**DOI:** 10.1007/s11306-017-1255-2

**Published:** 2017-09-20

**Authors:** K. Sattler, M. Behnes, C. Barth, A. Wenke, B. Sartorius, I. El-Battrawy, K. Mashayekhi, J. Kuschyk, U. Hoffmann, T. Papavasiliu, C. Fastner, S. Baumann, S. Lang, X. Zhou, G. Yücel, M. Borggrefe, I. Akin

**Affiliations:** 1 First Department of Medicine, Faculty of Medicine Mannheim, University Medical Centre Mannheim (UMM), University of Heidelberg, Theodor-Kutzer-Ufer 1-3, 68167 Mannheim, Germany; 2 Clinic of Cardiology and Angiology II, Universitäts-Herzzentrum Freiburg–Bad Krozingen, Bad Krozingen, Germany; 3 DZHK (German Center for Cardiovascular Research), Partner Site, Heidelberg-Mannheim, Mannheim, Germany

**Keywords:** LAA occlusion device, Left atrial appendage, Left atrial appendage closure, Atrial fibrillation, Metabolomics, SDMA, Sarcosine

## Abstract

**Background:**

Left atrial appendage (LAA) closure (LAAC) by implantation of an occlusion device is an established cardiac intervention to reduce risk of stroke while avoiding intake of oral anticoagulation medication during atrial fibrillation. Cardiac interventions can alter local or systemic gene and protein expression. Effects of LAAC on systemic metabolism have not been studied yet.

**Objectives:**

We aimed to study the effects of interventional LAAC on systemic metabolism.

**Methods:**

Products of glycolysis, tricarboxylic acid and urea metabolism were analyzed by ESI-LC-MS/MS and MS/MS using the AbsoluteIDQ™ p180 Kit in plasma of 44 patients undergoing successful interventional LAAC at baseline (T0) and after 6 months (T1).

**Results:**

During follow up, plasma concentrations of several parameters of glycolysis and tricarboxylic acid cycle (TCA) and urea metabolism increased (alanine, hexose, proline, sarcosine), while others decreased (aspartate, glycine, SDMA, serine). Multivariate linear regression analysis showed that time after interventional LAAC was an independent predictor for metabolite changes, including the decrease of SDMA (beta −0.19, p < 0.01) and the increase of sarcosine (beta 0.16, p < 0.01).

**Conclusions:**

Successful interventional LAAC affects different pathways of the metabolome, which are probably related to cardiac remodeling. The underlying mechanisms as well as the long term effects have to be studied in the future.

**Electronic supplementary material:**

The online version of this article (doi:10.1007/s11306-017-1255-2) contains supplementary material, which is available to authorized users.

## Introduction

Atrial fibrillation (AF) is the most common arrhythmia, affecting up to 2% of the population in Western countries (European Heart Rhythm et al. 2010). Cardiovascular events related to atrial fibrillation are stroke, death, worsening of left ventricular function, increase of vascular dementia and cognitive impairment, increase of hospitalization rate and reduction of quality of life (Kirchhof et al. 2016). To overcome the increased risk of stroke related to thromboembolic events out of the left atrium or the left atrial appendage, oral anticoagulation (OAC) guided by the CHA_2_DS_2_-VASc score is recommended (Kirchhof et al. 2016). The exclusion of the left atrial appendage (LAA) by LAA occlusion devices is a possible treatment strategy in patients with contraindications for OAC or with stroke while being treated with OAC (Kirchhof et al. 2016). The LAA is the main pressure sensor of the chambers of the left heart and secrets atrial natriuretic peptide (ANP) (Hara et al. 2009). Recently, metabolic changes in atrial tissue of patients with permanent AF compared to tissue of patients in sinus rhythm were identified by metabolomics studies (Mayr et al. 2008). They comprised metabolites of the utilization of ketone bodies and of the tricarboxylic acid (TCA) cycle as well as glycolytic enzymes (Mayr et al. 2008). The effects of different cardiac interventions either locally on gene or protein expression or systemically have been described several times (McManus et al. 2015; Navaravong et al. 2015; Huang et al. 2014; Xu et al. 2016; Nemutlu et al. 2015). In the current study, we tested whether successful interventional LAA closure (LAAC) would change the systemic metabolomic profile of patients with AF.

## Materials and methods

### Study population

The “Left Atrial Appendage Occlusion and Biomarker Evaluation” (LABEL) study (ClinicalTrials.gov Identifier: NCT02985463) is a single-center, prospective, hypothesis generating, observational non-randomized study including patients eligible for percutaneous LAAC according to the guidelines of the European Society of Cardiology (European Heart Rhythm et al. 2010). All patients presented with non-valvular AF, a CHA2DS2-Vasc score ≥2, a HAS-Bled score ≥3, and a contraindication for the therapy with oral anticoagulants, such as major or recurring bleeding. Exclusion criteria were age <18 years, congestive heart failure classified as NYHA IV, catheter ablation of AF within 30 days prior to planned intervention, myocardial infarction within the last 3 months, atrial septum defect or implanted devices for atrial septal defect (ASD), mechanical heart valves, status after heart transplant, symptomatic carotid artery stenosis, transient ischemic attack or stroke within 3 months, existing or planned pregnancy, acute infection, or detection of intracardiac thrombus at the day of planned implantation. The study was carried out according to the declaration of Helsinki and was approved by the medical ethics commission II of the Faculty of Medicine Mannheim, University of Heidelberg, Germany. Written informed consent was obtained from all participants or their respective legal representative.

### Interventional LAAC

Interventional LAAC was performed using either the Watchman (Boston Scientific, Marlbrough, MA, USA) or Amplatzer Amulet (St. Jude Medical, St. Paul, MN, USA) device. Successful LAAC was confirmed by transoesophageal echocardiography (TEE) during index procedure, as well as at mid-term follow-up by TEE and cardiac computed tomography angiography (CCTA).

### Sample preparation

Blood samples were taken by venous puncture within 24 h prior to cardiac intervention (T0) and mid-term follow-up, T1). At the time of blood sampling, all patients were clinically stable and without symptoms or clinical aspect of angina pectoris, dyspnoea, peripheral oedema, acute renal failure, exacerbation of chronic obstructive pulmonary disease or acute infection. Patients were in non-fasting state at time of blood drawing. At T1, blood was drawn after verification of successful closure of the LAA, as described above. Venous blood samples were collected from each patient into EDTA monovettes^®^ (Sarstedt, Germany) and centrifuged (2500 g, 10 min, 20 °C). The plasma was aliquotted, snap frozen in liquid nitrogen and stored at −80°. Processing was performed within two hours after blood extraction. Samples of the whole study population of T0 and T1 were shipped on dry ice to Biocrates (BIOCRATES Life Sciences AG, Innsbruck, Austria) for further analysis. Storage was continued at −80 °C. Sample preparation was done as described previously (Siskos et al. 2017). During further processing until measurement, samples were handled on ice.

### Metabolite analysis

A targeted metabolomics approach based on electrospray ionization liquid chromatography–mass spectrometry (ESI-LC-MS/MS) and MS/MS measurements was performed using the AbsoluteIDQ™ p180 Kit (BIOCRATES Life Sciences AG, Innsbruck, Austria). The assay allows simultaneous quantification of in total 188 metabolites out of 10 μL plasma samples, including amino acids, biogenic amines, glycerophospholipids, sphingolipids and the sum of hexoses. Analyses were carried out on a QTRAP 4000 System (Sciex Deutschland GmbH, Darmstadt, Germany) and a Thermo TSQ (ThermoFisher Scientific, Waltham, USA). Metabolite measurements were carried out as described previously (Siskos et al. 2017). All samples were prepared and measured simultaneously, being randomly allotted to two different assay plates. For the evaluation of metabolite concentrations, internal standards served as a reference. Metabolites were defined as MSI level I metabolites. BIOCRATES MetIDQ™ software was used for the processing and technical validation of the metabolite data. Sample processing and further analyses were performed by technicians blinded for patients’ characteristics.

### Statistical analysis

To exclude metabolites of which concentration values were below LOD, a general cleaning of the data set based on an 80% rule was performed. This manuscript focusses on glycolysis, tricarboxylic acid and urea cycle. Results of metabolites of different pathways will be presented elsewhere. Metabolites of the pathways in focus excluded as based on the 80% rule are listed in detail in Supplemental Table 1. Remaining values in the data set were then imputed applying a logspline imputation method. The resulting data set was log2 transformed (Guida et al. 2016; Kooperberg 1992). Principal Component Analysis (PCA), Partial Least Squares Discrimination Analysis (PLS-DA) and Hierarchical cluster analysis (HCA) were used as supervised and unsupervised multivariate approaches (Worley and Powers 2013). To compare significant differences, data were subjected to a student’s *t* test or repeated measures ANOVA (rANOVA). To control the false-discovery-rate (FDR) during multiple comparisons, an adjusted p value (Benjamini-Hochberg correction) was additionally calculated (Benjamini 1995). A regression analysis based on a linear mixed effect model was applied for the evaluation of dependency of significant metabolite change on clinical factors which had shown changes in metabolite concentration between T0 and T1 (gender, age, diabetes mellitus, body mass index, left ventricular ejection fraction, creatinine, pro-B natriuretic peptide). For continuous variables, the median was chosen to dichotomize the data. Statistical analysis was performed using R-Studio (RStudio and RStudio 2015).

## Results

### Patients’ characteristics

Altogether, 44 patients were treated by successful interventional LAAC. Median age was 78 years. 77% of patients had experienced a major bleeding event prior to the intervention. Median CHA2DS2-VASc score and HAS-BLED score was 4 (interquartile range (IQR) 3–5 and 3–4.3, respectively). Further demographic and clinical characteristics of the patients are shown in Table 1.

**Table 1:** Baseline characteristics of 44 patients with successful interventional left atrial appendage closure

Demographic factors	
Male	30 (68.2)
Age, years (range)	78 (43.0–87.0)
BMI, kg/m^2^	28.1 (24.7–32.7)
NTpro-BNP, ng/l	975.3 (455.2–1429.0)
Cardiovascular risk factors, n (%)	
Hypertension	42 (95.4)
Diabetes mellitus	16 (36.7)
Hypercholesterinemia	22 (50.0)
Medical history, n (%)	
Atrial fibrillation	
Paroxysmal	24 (54.5)
Persistent	6 (13.5)
Permanent	14 (31.8)
LV-EF	
Normal (>55%)	34 (77.2)
Mild	4 (9.1)
Moderate	4 (9.1)
Severe	2 (4.5)
Prior PVI	4 (9.1)
TIA	3 (6.8)
Stroke	7 (15.9)
Coronary artery disease	25 (56.8)
Prior myocardial infarction	10 (22.7)
Heart failure	10 (22.7)
Peripheral vascular disease	4 (9.1)
Chronic kidney disease	18 (40.1)
Creatinine, mg/dl (IQR)	1.05 (0.9–1.3)
MDRD-GFR, ml/min (IQR)	65.5 (52.7–79.7)
Chronic liver disease	3 (6.8)
Prior bleeding	34 (77.3)
CHA_2_DS_2_-VASc score (IQR)	4 (3.0–5.0)
HAS-BLED score (IQR)	4 (3.0–4.3)
Medication	
Beta-blockers	28 (63.6)
Diuretics	32 (72.7)
ACE-inhibitors/ARBs	20 (45.5)
Aldosteron receptor antagonist	2 (4.5)
Events during follow-up, n (%)	
Acute myocardial infarction	1 (2.3)
Stroke	0 (0)
Pulmonary embolism	1 (2.3)
Bleeding according to BARC score	8 (18.2)
Type 1	1 (2.3)
Type 2	5 (11.4)
Type 3a	2 (4.5)
≥Type 3b	0
Rehospitalization	24 (54.5)

Values are given as median (25th and 75th percentiles) or total numbers (percentage)

*ACE* angiotensin converting enzyme, *AF* atrial fibrillation, AMI acute myocardial infarction, *ARB* angiotensin receptor blocker, *BARC* bleeding academic research consortium, *BMI* body mass index, *IQR* interquartile range, *LV-EF* left ventricular ejection fraction, *NTpro-BNP* N terminal pro-B type natriuretic peptide, *PVI* pulmonary vein isolation, *TIA* transient ischemic attack, *MDRD*-*GFR* modification of diet in renal disease-glomerular filtration rate

### Metabolite changes after LAAC

Metabolic alterations after LAAC were measured in plasma samples collected prior to intervention (T0) and after 6 months (T1). Results of the PCA analysis were not significant different between both groups (data not shown).

**Fig. 1:**
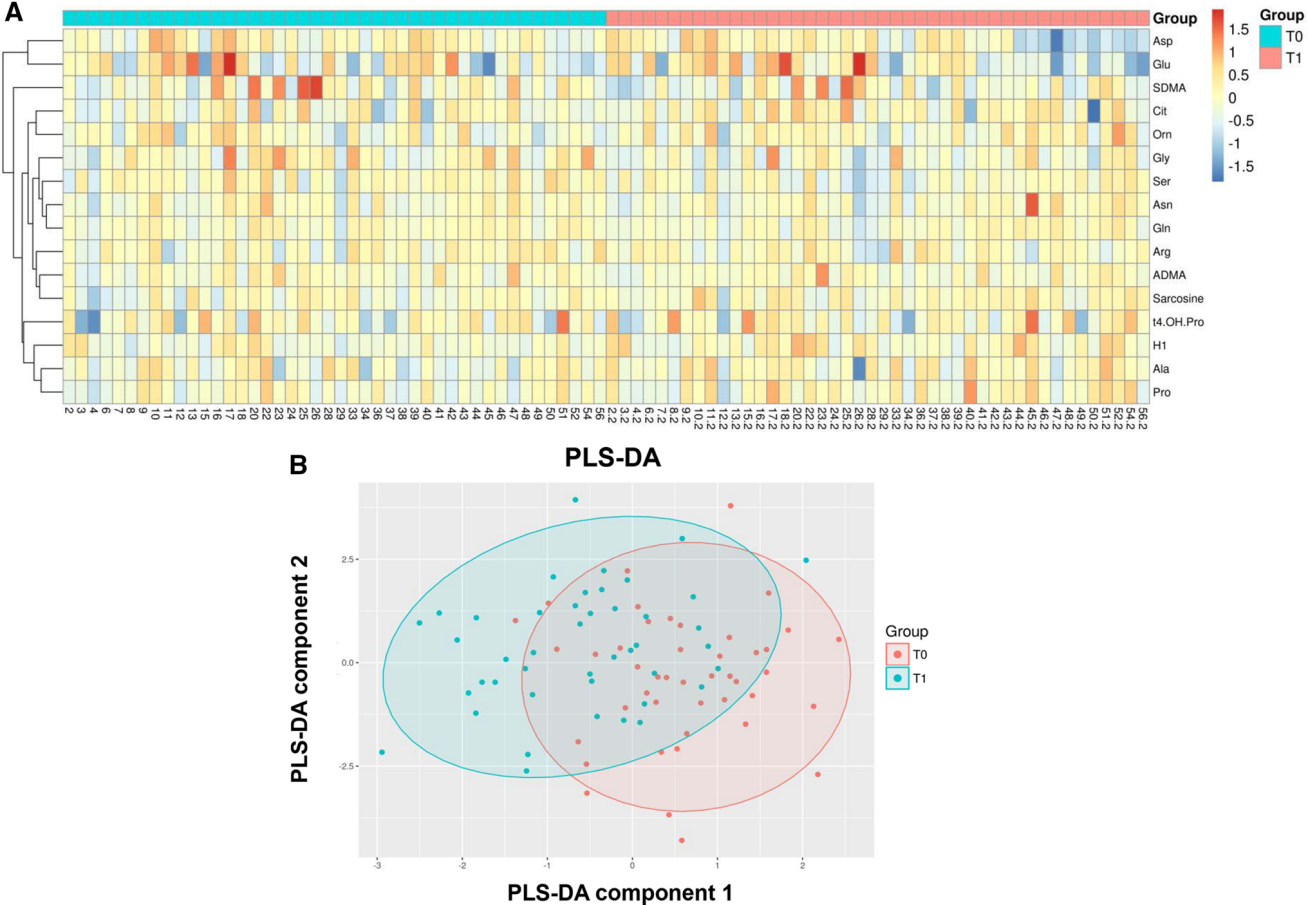
**a** Hierarchical cluster analysis of metabolite concentrations at T0 versus T1 of the 44 study patients. Plasma samples were measured by mass spectrometry (ESI-LC-MS/MS and MS/MS) with an Absolute*IDQ*™ p180. *Numbers* indicate the individual sample numbering at intervention (*single number*) and follow up (extension “0.2”). *ADMA* asymmetric dimethylarginine, *Ala* alanine, *Arg* arginine, *Asn* asparagine, *Asp* asparate, *Cit* citrulline, *Gln* glutamine, *Glu* glutamate, *Gly* glycine, *Orn* ornithin, *Pro* proline, *SDMA* symmetric dimethylarginine, *Ser* serine, *t4-OH-Pro* trans 4-hydroxy-proline. **b** Score plot of the PLS-DA analysis comparing the log transformed datasets of metabolite concentrations at T0 (*red*) versus T1 (*blue*). The *areas* represent the 95% confidence interval. R^2^X = 60.1%, R^2^Y = 23.1%

**Fig. 2:**
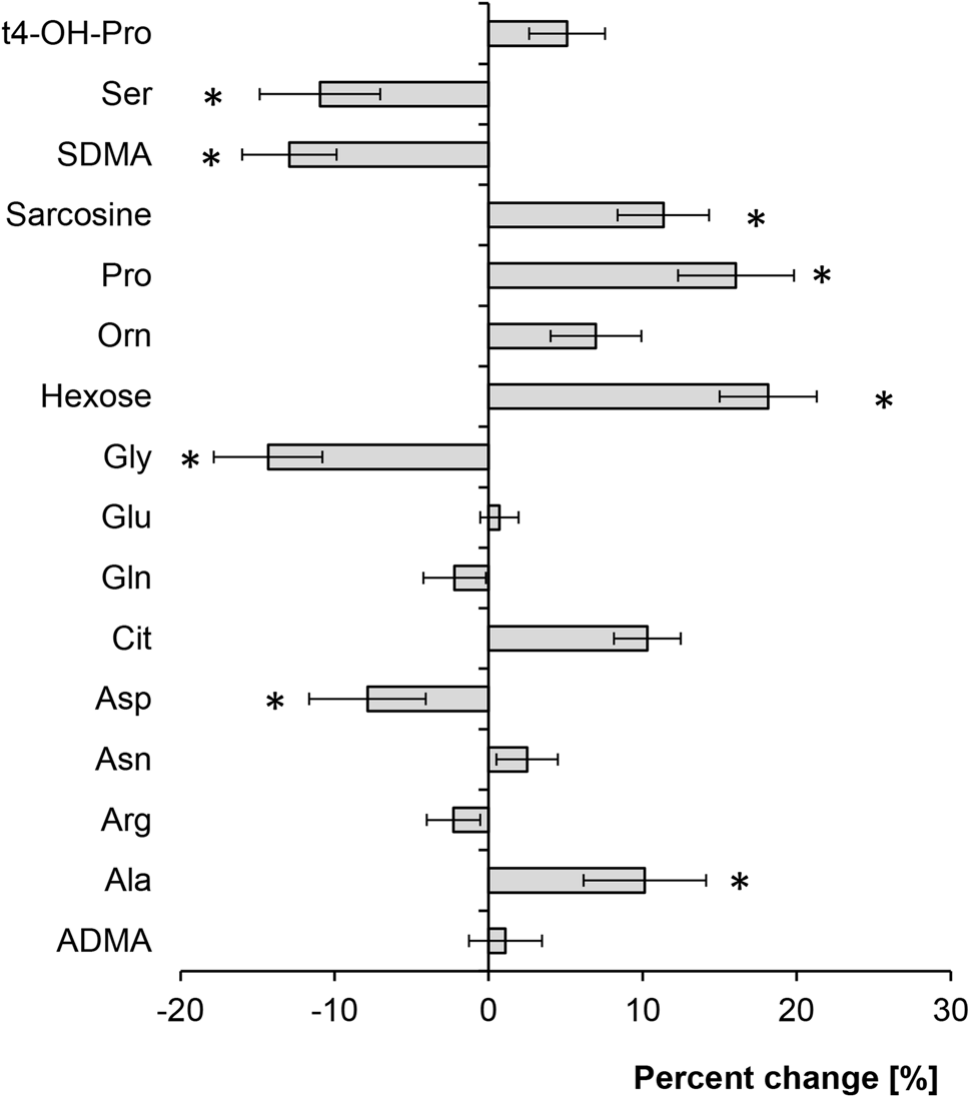
Effect of LAAC on the metabolome. Mean percent change of the concentrations of different metabolites during follow up. For abbreviations, see legend of Fig. 1a. *p < 0.05 versus concentration at baseline

The Hierarchical cluster analysis (HCA) showed that the levels of several metabolites were different at T0 and T1 in some, but not all patients, with high inter-individual differences (Fig. 1a). In the PLS-DA analysis partial separation could be detected between samples before LAAC and at follow-up (Fig. 1b). Altogether, several metabolites increased significantly over time (alanine, hexose, proline, sarcosine), while others decreased (aspartate, glycine, SDMA, serine). The quantitative data are shown in Table 2, and the percentages of change of the metabolites’ concentrations are presented in Fig. 2. Patients’ medication targeting volume status, electrolytes or sympathetic activation, such as diuretics, beta-blockers or ACE-inhibitors or aldosterone receptor antagonists, was not changed in type or dosage during follow-up.

**Table 2:** Concentrations and mean percent change of different metabolites before (T0) and 6 months after (T1) successful interventional LAAC

Metabolite	T0 (µM)	T1 (µM)	Percent change	p value
Asymmetric dimethylarginine (ADMA)	0.55 ± 0.14	0.55 ± 0.15	1.09 ± 2.37	0.87
Alanine (Ala)	307.05 ± 102.32	338.19 ± 107.43	10.14 ± 3.98	**0.047**
Arginine (Arg)	66.33 ± 17.12	64.82 ± 17.00	−2.28 ± 1.74	0.66
Asparagine (Asn)	39.13 ± 10.18	40.11 ± 14.80	2.50 ± 2.00	0.85
Aspartate (Asp)	5.34 ± 1.70	4.92 ± 1.89	−7.87 ± 3.78	**0.049**
Citrulline (Cit)	33.77 ± 11.39	37.25 ± 12.65	10.31 ± 2.17	0.09
Glutamine (Gln)	661.5 ± 109.10	646.89 ± 120.87	−2.21 ± 2.02	0.31
Gutamate (Glu)	86.54 ± 51.92	87.14 ± 51.72	0.69 ± 1.24	0.85
Glycine (Gly)	263.89 ± 104.57	226.11 ± 85.56	−14.32 ± 3.52	< **0.001**
Hexose	6703.41 ± 1754.89	7920.14 ± 2478.32	18.15 ± 3.15	< **0.001**
Ornithin (Orn)	80.63 ± 26.71	86.25 ± 27.88	6.97 ± 2.94	0.13
Proline (Pro)	203.82 ± 54.85	236.55 ± 79.86	16.06 ± 3.76	**0.002**
Sarcosine	2.29 ± 0.54	2.55 ± 0.59	11.35 ± 2.97	**0.002**
Symmetric dimethylarginine (SDMA)	0.85 ± 0.46	0.74 ± 0.37	−12.94 ± 3.07	< **0.001**
Serine (Ser)	106.98 ± 27.49	95.27 ± 24.86	−10.95 ± 3.91	**0.007**
Trans 4-hydroxy-Proline (t4-OH-Pro)	11.97 ± 5.22	12.58 ± 5.54	5.10 ± 2.46	0.42

Metabolites appear in alphabetical order. Data are presented as mean concentration ± standard deviation

Statistical significance p < 0.05 is highlighted in bold

### Effects of clinical and demographic factors

Subsequently, we tested whether different clinical and epidemiological factors would affect metabolite concentration. Concentrations of metabolites differed in subgroups defined by clinical or demographic factors (gender, age, diabetes mellitus, body mass index, left ventricular ejection fraction, creatinine, NTpro-BNP). The results of the repeated measurement ANOVAs are shown in detail in the supplemental tables 2 to 8 for each subgroup.

### Time after LAAC is an independent predictor for metabolite change

**Fig. 3:**
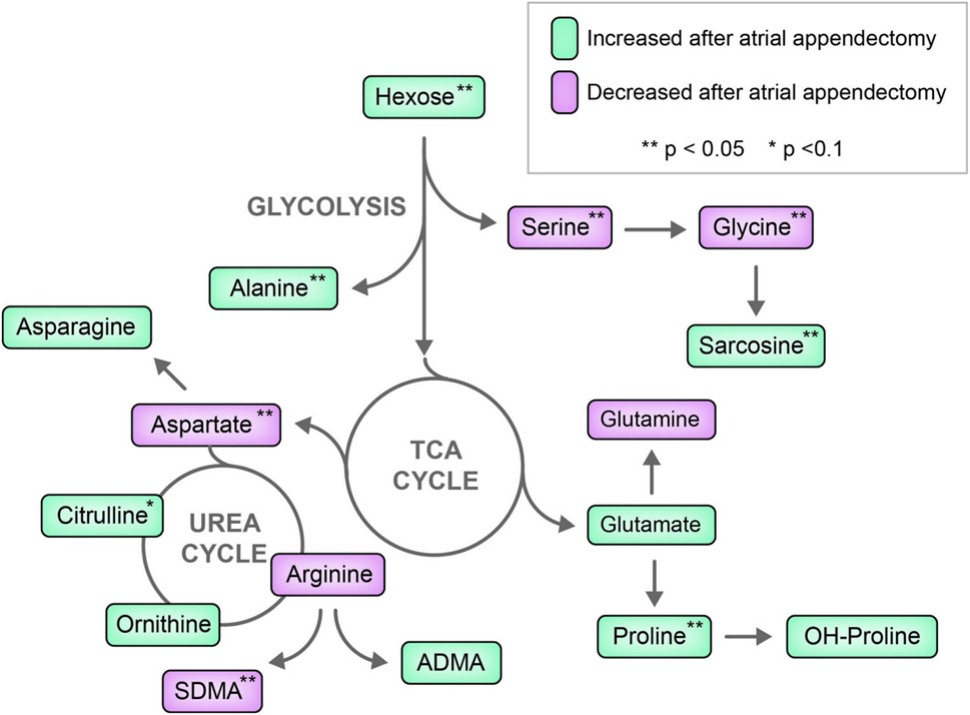
Pathways and metabolite concentrations affected by LAAC as assessed by mixed linear regression analysis. *Green labeling* positive association of metabolites with time after follow up. *Purple labeling* negative association of metabolites with time after follow up. *p < 0.1, **p < 0.05

Multivariate regression analysis showed that the parameter “time after implantation” was associated with changes of plasma concentration of eight different metabolites (positive association with concentrations of hexose, proline, alanine, sarcosine; negative association with concentrations of SDMA, glycine, serine, aspartate, Table 3), while the other independent variables (as defined in the previous paragraph) had only inconsistent influence on metabolite levels (Supplemental Table 9). Figure 3 depicts the results of the regression analysis in context of the affected metabolic cycles (Fig. 3).

**Table 3:** Association of the independent parameter “T0 versus T1” with metabolite concentrations in the linear mixed model regression analysis

Rank	Metabolite	Beta	SE	T value	CI (2.5%)	CI (97.5%)	FDR	p value
1	Hexose	0.21	0.05	4.50	0.12	0.31	<0.01	<**0.01**
2	Symmetric dimethylarginine (SDMA)	−0.19	0.05	−4.04	−0.28	−0.10	<0.01	<**0.01**
3	Glycine (Gly)	−0.21	0.06	−3.63	−0.32	−0.10	<0.01	<**0.01**
4	Proline (Pro)	0.18	0.05	3.39	0.08	0.29	<0.01	<**0.01**
5	Sarcosine	0.16	0.05	3.27	0.06	0.26	<0.01	<**0.01**
6	Serine (Ser)	−0.17	0.06	−2.84	−0.29	−0.05	0.02	<**0.01**
7	Alanine (Ala)	0.14	0.07	2.05	0.004	0.27	0.10	<**0.05**
8	Aspartate (Asp)	−0.17	0.08	−2.02	−0.22	−0.003	0.10	<**0.05**
9	Citrulline (Cit)	0.13	0.07	1.74	−0.02	0.28	0.16	0.09
10	Ornithin (Orn)	0.10	0.07	1.54	−0.03	0.24	0.21	0.13
11	Glutamine (Gln)	−0.04	0.04	−1.02	−0.11	0.04	0.46	0.31
12	trans 4-hydroxy-Proline (t4-OH-Pro)	0.07	0.09	0.81	−0.11	0.26	0.57	0.42
13	Arginine (Arg)	−0.03	0.07	−0.45	−0.16	0.11	0.81	0.66
14	Asparagine (Asn)	0.01	0.06	0.19	−0.10	0.12	0.87	0.85
15	Gutamate (Glu)	0.02	0.12	0.19	−0.21	0.26	0.87	0.85
16	Asymmetric dimethylarginine (ADMA)	0.01	0.03	0.16	−0.05	0.06	0.87	0.87

*CI* confidence interval, *FDR* false discovery rate, *SE* standard error

Statistical significance p < 0.05 is highlighted in bold

## Discussion

In this prospective, hypothesis generating, observational study, we show that successful interventional LAAC under conditions of AF induces changes of the plasma concentration of metabolites of all three components of nutrient processing, namely glycolysis, tricarboxylic acid and protein metabolism. The observed changes might mirror remodeling processes of the left atrium and ventricle ongoing after device implantation.

The atrial appendages are remnants from heart development and are structurally and anatomically divers from the atria (DeSimone et al. 2015). Structurally, within the appendages an epicardial and an endocardial layer can be distinguished. Of the LAA, the epicardial layer consists of myocytes as well as of cells of the conduction system, i.e. of Bachman’s bundle (DeSimone et al. 2015). The myocytes of the appendages resemble ventricular myocytes rather than atrial myocytes, and form comb-like trabeculae within the appendages (Al-Saady et al. 1999). The atrial appendages secrete atrial natriuretic peptide, thus being part of the fluid homeostasis of the body (DeSimone et al. 2015). They are connected to the autonomous nervous system and take part in heart rhythm regulation, probably via stretch sensors (Kappagoda et al. 1972). Furthermore, they play a major role in hemodynamics as they serve as a volume reservoir and add to the cardiac output by contraction (Stollberger et al. 2003). In addition, the appendages contain cardiac stem cells, which are able to differentiate into cardiomyocytes (Koninckx et al. 2013), a property recently used successfully for cardiac repair in an animal model of myocardial infarction (Fanton et al. 2015). During AF, LAA remodeling consisting of dilation, endocardial fibroelastosis and myocyte thinning was observed (DeSimone et al. 2015; Stollberger et al. 2003), while LAA contractility was greatly reduced, preparing the ground for thrombus formation (Pollick and Taylor 1991).

Metabolomics is “the study of the metabolic profile of small molecules in a biological organism” (Deidda et al. 2015). By applying different analytic techniques, such as nuclear magnetic resonance spectroscopy (NMR) or mass spectrometry (MS), metabolomics allows identifying and quantifying a high number of metabolites in a sample. Despite the known impairment of metabolism in patients with heart failure, both locally (Xu et al. 2016) as well systemically (Hunter et al. 2016), metabolomics studies were conducted only on atrial tissue but not on plasma samples of patients with AF so far (Mayr et al. 2008). Similarly, neither the effect of the termination of AF (either electrically or pharmaceutically) nor of the exclusion of LAA from the circulation by surgery or device implantation has been studied yet. When considering the role of the atrial appendages in hemodynamics and homeostasis, however, any intervention targeting these structures are likely to affect the systemic metabolome as well. Similar observations were made for example in the case of treatment of heart failure with cardiac synchronization therapy (Nemutlu et al. 2015).

In the current study, we used plasma samples to test whether any systemic changes occur after LAAC. We were able to demonstrate that several metabolites changed over time after the implantation of the device. Of course, our data are purely observational and do not allow concluding any pathophysiologic mechanisms. In addition, the measurements were done in plasma while the metabolic cycles studied take place intracellularly, which does only imperfectly allow drawing conclusions on the precise mechanisms modified by LAA exclusion from the circulation. Nevertheless, one could speculate that “occluding” the LAA from the circulation by LAAC induces changes in morphology and hemodynamics of the LAA, which secondarily have an effect on cardiac, and subsequently, on systemic hemodynamics. A further explanation might be an induction of a change of LAA endocrine function by the implanted device by excluding the LAA from volume overload and tissue re-organisation during AF.

From our data, we would like to discuss two distinct results further, due to their possible or already described connection to the cardiovascular system: (1) We found an increase of sarcosine paralleled by a decrease of its precursor, glycine. Recently, an inverse association between glycine plasma levels and the occurrence of myocardial infarction were demonstrated (Ding et al. 2015). Sarcosine (*N*-methylglycine), a glycine transporter-I (GT-I)-inhibitor, seems to be an important factor for tissue proliferation, as it was found to be relevant for angiogenesis (Sudhakaran et al. 2014) and cell cycle progression during tumor growth (Sreekumar et al. 2009). Furthermore, by inhibiting GT-I, sarcosine displays antidepressant and anti-psychotic effects and is indeed considered a tool to treat depression or schizophrenia (Mathew 2013). The role of sarcosine for cardiac tissue or cardiac metabolism has not been studied so far. However, it might be in part responsible for any restructuring of atrial tissue after LAAC, and might also play a role for linking cardiovascular diseases and psychiatric conditions. (2) We found a decrease of the concentration of symmetric dimethylarginine (SDMA) over time. SDMA is a byproduct of the citrulline-NO-cycle, the recycling pathway of NO production which takes place in many different cell types (Mori and Gotoh 2000). SDMA was reported to have pro-inflammatory effects on cells of the immunosystem and to increase reactive oxygen species (Tain and Hsu 2017). Being part of the urea cycle, its plasma levels correlate with kidney dysfunction (Tain and Hsu 2017), and its role for HDL dysfunction in patients suffering from chronic kidney disease was described only recently (Zewinger et al. 2017). However, the precise role of SDMA in the cardiovascular system is unclear so far. Interestingly, a recently published, large population-based study including nearly 5000 individuals with and without AF found a positive correlation between SDMA concentration in plasma and both the dimensions of the left atrium and the left ventricular mass (Ramuschkat et al. 2016). So far, there are no data of the effects of LAAC on the dimensions of the left heart. However, based on our findings one can hypothesize that occluding the LAA affects the size of the left atrium as well as the hemodynamic load of the left ventricle, as mirrored by SDMA concentration. The remodeling mechanisms responsible for such changes have to be defined yet.

Taken together, our study underlines the importance of further research to study the effects of this specific cardiac device therapy on cardiac remodeling and on the systemic metabolome.

### Study limitations

This was a prospective, hypothesis generating, observational study which did not include a control group of patients with AF but without LAAC, or a group of patients without AF. The change of the molecular pathways, their time course and the biologic significance need to be established in future studies. The standard deviation of several metabolite concentrations was very high. This could mirror inter-individual differences between the patients, and might be in part due to low sample number. Including a larger study population would show whether these assumptions are correct. We cannot rule out an influence on patients’ medication on the time course of metabolites. As patients´ cardiovascular medication did not change in type or dosage, the imposed effect had to be a constant one, starting with the beginning of drug intake, and being observed in patients without LAAC as well. The answer to this question could be derived from a control group of AF patients, as discussed above. This manuscript focusses on metabolites of glycolysis, tricarboxylic acid and urea cycle. Further metabolites of different pathways were beyond the scope of the present study. Further upcoming studies will evaluate metabolites apart from the presented ones.

### Clinical perspectives

Interventional LAAC is an established means to prevent atrial fibrillation-associated thrombembolism. The current study shows effects on metabolomic pathways, in part related to cardiac remodeling. Cardiac structural changes, and mechanisms and long-term effects of the observed metabolomic changes have to be studied in the future.

## Electronic supplementary material

Below is the link to the electronic supplementary material.

Supplementary material 1 (DOCX 57 KB)
