# Correction to: Occlusion of left atrial appendage affects metabolomic profile: focus on glycolysis, tricarboxylic acid and urea metabolism

**DOI:** 10.1007/s11306-017-1301-0

**Published:** 2018-01-12

**Authors:** K. Sattler, M. Behnes, C. Barth, A. Wenke, B. Sartorius, I. El-Battrawy, K. Mashayekhi, J. Kuschyk, U. Hoffmann, T. Papavasiliu, C. Fastner, S. Baumann, S. Lang, X. Zhou, G. Yücel, M. Borggrefe, I. Akin

**Affiliations:** 1 First Department of Medicine, Faculty of Medicine Mannheim, University Medical Centre Mannheim (UMM), University of Heidelberg, Theodor-Kutzer-Ufer 1-3, 68167 Mannheim, Germany; 2 Clinic of Cardiology and Angiology II, Universitäts-Herzzentrum Freiburg–Bad Krozingen, Bad Krozingen, Germany; 3 DZHK (German Center for Cardiovascular Research), Partner Site, Heidelberg-Mannheim, Mannheim, Germany

## Correction to: Metabolomics (2017) 13:127 10.1007/s11306-017-1255-2

The article Occlusion of left atrial appendage affects metabolomic profile: focus on glycolysis, tricarboxylic acid and urea metabolism, written by K. Sattler, M. Behnes, C. Barth, A. Wenke, B. Sartorius, I. El-Battrawy, K. Mashayekhi, J. Kuschyk, U. Hoffmann, T. Papavasiliu, C. Fastner, S. Baumann, S. Lang, X. Zhou, G. Yücel, M. BorggrefeI, Akin, was originally published Online First without open access. After publication in volume [13], issue [11], Citation ID [127] the author decided to opt for Open Choice and to make the article an open access publication. Therefore, the copyright of the article has been changed to © The Author(s) [2017] and the article is forthwith distributed under the terms of the Creative Commons Attribution 4.0 International License (http://creativecommons.org/licenses/by/4.0/), which permits use, duplication, adaptation, distribution and reproduction in any medium or format, as long as you give appropriate credit to the original author(s) and the source, provide a link to the Creative Commons license and indicate if changes were made. The original article has been corrected.

