# Supplementary material for: Occlusion of left atrial appendage affects metabolomic profile: focus on glycolysis, tricarboxylic acid and urea metabolism
Source: Metabolomics. 2017 Sep 20;13(11):127. doi: 10.1007/s11306-017-1255-2 (PMC5772135; doi:10.1007/s11306-017-1255-2)
Supplement: Supplementary file 1 — Supplementary material 1 (DOCX 57 KB) [file 11306_2017_1255_MOESM1_ESM.docx]

**Supplement**

**Occlusion of Left Atrial Appendage Affects Metabolomic Profile: Focus on Glycolysis, Tricarboxylic acid and Urea Metabolism**

Sattler K. ^1^*, Behnes M. ^1^*, Barth C. ^1^, Wenke A. ^1^, Sartorius B. ^1^, Saleh, A. ^1^, Mashayekhi K. ^2^, Ansari U. ^1^, Hoffmann U. ^1^, Akin M. ^3^, Fastner C. ^1^, Rusnak J. ^1^, Lang S. ^1^, Zhou X. ^1^, Yücel G. ^1^, Borggrefe M. ^1,4^, Akin I. ^1,4^.

**both authors contributed equally to the study.*

From the First Department of Medicine, Faculty of Medicine, University Medical Centre Mannheim (UMM), University of Heidelberg, Mannheim, Germany

1 - First Department of Medicine, Faculty of Medicine, University Medical Centre Mannheim (UMM), University of Heidelberg, Mannheim, Germany.

2 - Universitäts-Herzzentrum Freiburg – Bad Krozingen, Clinic of Cardiology and Angiology II, Bad Krozingen, Germany

3 - Department of Cardiology and Angiology, Hannover Medical School, Hannover, Germany

4 - DZHK (German Center for Cardiovascular Research), Partner Site, Heidelberg-Mannheim, Mannheim, Germany.

**Corresponding author**

Michael Behnes, MD

First Department of Medicine

University Medical Centre Mannheim (UMM)

Faculty of Medicine Mannheim

University of Heidelberg

Theodor-Kutzer-Ufer 1-3

68167 Mannheim, Germany

Phone: +49-6201-383-2512

Fax: +49-6201-383-2012

e-mail: Michael.Behnes@umm.de

**Supplemental Tables.**

| **Supplemental Table 1. Amnio acids and biogenic amines excluded from further analyses due to levels of detection below 80%.** |
| --- |
| Acetyl-ornithin |
| Carnosine |
| Dihydroxyphenylalanine |
| Dopamine |
| Histamine |
| Methioninesulfoxide |
| Nitro-tyrosine |
| Phenylethylamine |
| Serotonin |
| Spermidine |
| Spermine |
| C4-hydroxy-proline |
|  |

| **Supplemental Table 2. Repeated measures ANOVA of the metabolite concentrations grouped by the variables “T0 vs. T1” and “Gender”.** | | | | | | | |
| --- | --- | --- | --- | --- | --- | --- | --- |
| **Metabolite** | **T0** | | **T1** | |  | | |
|  | **Female (n=14)** | **Male (n=30)** | **Female (N=14)** | **Male (N=30)** | **FDR** | **P value** | |
| Hexose | 6685.93 ± 1862.14 | 6711.57 ± 1543.98 | 8043.07 ± 2929.42 | 7862.77 ± 2234.57 | 0.38 | | **0.02** |
| Pro | 188.00 ± 35.86 | 211.20 ± 58.86 | 220.93 ± 87.72 | 243.83 ± 74.81 | 0.38 | | 0.06 |
| Sarcosine | 2.18 ± 0.62 | 2.33 ± 0.50 | 2.48 ± 0.67 | 2.58 ± 0.55 | 0.38 | | 0.07 |
| Asp | 4.96 ± 1.62 | 5.51 ± 1.68 | 4.26 ± 1.67 | 5.23 ± 1.91 | 0.62 | | 0.28 |
| Gly | 334.50 ± 128.52 | 230.93 ± 70.79 | 265.14 ± 119.77 | 207.90 ± 54.78 | 0.62 | | 0.28 |
| Ala | 279.00 ± 111.59 | 320.13 ± 91.10 | 322.50 ± 107.81 | 345.51 ± 106.46 | 0.62 | | 0.29 |
| SDMA | 0.93 ± 0.43 | 0.81 ± 0.46 | 0.88 ± 0.38 | 0.67 ± 0.35 | 0.62 | | 0.29 |
| Orn | 77.77 ± 28.00 | 81.96 ± 26.23 | 76.28 ± 23.01 | 90.90 ± 28.72 | 0.62 | | 0.40 |
| Ser | 115.21 ± 33.13 | 103.14 ± 23.17 | 88.87 ± 22.23 | 98.25 ± 25.45 | 0.62 | | 0.41 |
| Asn | 40.19 ± 11.32 | 38.63 ± 9.55 | 42.43 ± 22.91 | 39.03 ± 8.52 | 0.62 | | 0.42 |
| Gln | 709.00 ± 115.05 | 639.33 ± 98.70 | 670.93 ± 122.10 | 635.67 ± 118.63 | 0.62 | | 0.45 |
| Cit | 38.52 ± 12.10 | 31.55 ± 10.46 | 38.58 ± 13.58 | 36.63 ± 12.14 | 0.62 | | 0.48 |
| t4.OH.Pro | 11.10 ± 5.18 | 12.38 ± 5.06 | 12.82 ± 7.09 | 12.48 ± 4.63 | 0.62 | | 0.50 |
| Arg | 68.93 ± 18.14 | 65.12 ± 16.76 | 64.37 ± 20.62 | 65.03 ± 15.01 | 0.79 | | 0.69 |
| Glu | 80.82 ± 64.74 | 89.20 ± 44.98 | 70.11 ± 37.53 | 95.09 ± 55.38 | 0.81 | | 0.80 |
| ADMA | 0.58 ± 0.20 | 0.53 ± 0.10 | 0.59 ± 0.21 | 0.53 ± 0.10 | 0.81 | | 0.81 |
| Data are presented as mean concentration [µM] ± standard deviation. FDR, false discovery rate. ADMA, asymmetric dimethylarginine; Ala, alanine; Arg, arginine; Asn, asparagine; Asp, asparate; Cit, citrulline; Gln, glutamine; Glu, glutamate; Gly, glycine; Orn, ornithin; Pro, proline; SDMA, symmetric dimethylarginine; Ser, serine; t4-OH-Pro; trans 4-hydroxy-proline | | | | | | | |

| **Supplemental Table 3. Repeated measures ANOVA of the metabolite concentrations grouped by the variables “T0 vs. T1” and “BMI < vs. > 25kg/m^2^”.** | | | | | | | |
| --- | --- | --- | --- | --- | --- | --- | --- |
| **Metabolite** | **T0** | | **T1** | |  | | |
|  | **BMI > 25 kg/m^2^ (n=29)** | **BMI < 25 kg/m^2^ (n=15)** | **BMI > 25 kg/m^2^ (n=29)** | **BMI < 25 kg/m^2^ (n=15)** | **FDR** | **P value** | |
| Asp | 5.43 ± 1.69 | 5.15 ± 1.65 | 5.05 ± 1.68 | 4.68 ± 1.79 | 0.30 | | **0.02** |
| Pro | 201.66 ± 52.43 | 208.00 ± 55.95 | 231.28 ± 58.86 | 246.73 ± 105.28 | 0.30 | | **0.05** |
| Hexose | 6926.17 ± 1744.11 | 6272.73 ± 1357.25 | 8315.34± 1543.98 | 7156.07 ± 1983.43 | 0.30 | | 0.09 |
| t4.OH.Pro | 12.28 ± 4.52 | 11.37 ± 6.11 | 12.76± 5.06 | 12.25 ± 6.75 | 0.30 | | 0.09 |
| Sarcosine | 2.30 ± 0.53 | 2.25 ± 0.58 | 2.53 ± 0.50 | 2.59 ± 0.60 | 0.30 | | 0.12 |
| Ser | 106.92 ± 29.34 | 107.08 ± 22.96 | 93.35 ± 23.17 | 98.97 ± 24.58 | 0.30 | | 0.13 |
| Gly | 263.62 ± 110.41 | 264.40 ± 93.26 | 218.69 ± 70.79 | 240.47 ± 85.62 | 0.30 | | 0.17 |
| Orn | 85.42 ± 25.83 | 71.36 ± 26.43 | 89.63 ± 26.23 | 79.71 ± 31.07 | 0.30 | | 0.18 |
| Cit | 35.43 ± 12.13 | 30.55 ± 9.29 | 838.47 ± 10.46 | 34.89 ± 11.00 | 0.30 | | 0.19 |
| SDMA | 0.95 ± 0.51 | 0.65 ± 0.21 | 0.80 ± 0.46 | 0.61 ± 0.24 | 0.30 | | 0.19 |
| Ala | 303.45 ± 106.56 | 314.00 ± 85.27 | 320.15 ± 83.83 | 373.07 ± 135.72 | 0.31 | | 0.21 |
| Gln | 671.83 ± 106.86 | 641.53 ± 110.65 | 664.93 ± 98.70 | 612.00 ± 98.74 | 0.38 | | 0.29 |
| Arg | 66.62 ± 15.70 | 65.77 ± 20.02 | 64.72 ± 16.76 | 65.88 ± 21.90 | 0.39 | | 0.32 |
| ADMA | 0.57 ± 0.15 | 0.49 ± 0.07 | 0.57 ± 0.10 | 0.50 ± 0.08 | 0.62 | | 0.56 |
| Glu | 85.73 ± 52.83 | 88.10 ± 51.03 | 90.21 ± 44.98 | 81.21 ± 37.05 | 0.62 | | 0.58 |
| Asn | 40.39 ± 10.52 | 36.69 ± 8.97 | 38.66 ± 9.55 | 42.92 ± 21.46 | 0.93 | | 0.93 |
| Data are presented as mean concentration [µM] ± standard deviation. For abbreviations, see Supplemental Table 1. | | | | | | | |

| **Supplemental Table 4. Repeated measures ANOVA of the metabolite concentrations grouped by the variables “T0 vs. T1” and “age < vs. > 77years”.** | | | | | | | |
| --- | --- | --- | --- | --- | --- | --- | --- |
| **Metabolite** | **T0** | | **T1** | |  | | |
|  | **age >77years (n=23)** | **age <77years (n=21)** | **age >77years (n=23)** | **age < 77years (n=21)** | **FDR** | **P value** | |
| Cit | 34.85 ± 11.29 | 32.69 ± 11.65 | 39.19 ± 14.31 | 35.31 ± 10.38 | 0.52 | | **0.04** |
| Gly | 286.41 ± 95.06 | 241.36 ± 109.31 | 241.68 ± 69.97 | 210.55 ± 96.23 | 0.52 | | 0.06 |
| Sarcosine | 2.24 ± 0.48 | 2.33 ± 0.61 | 2.56 ± 0.56 | 2.54 ± 0.63 | 0.57 | | 0.13 |
| SDMA | 0.89 ± 0.44 | 0.81± 0.47 | 0.82 ± 0.43 | 0.66 ± 6.70 | 0.57 | | 0.14 |
| Pro | 208.23 ± 54.85 | 199.41 ± 52.25 | 255.91 ± 84.13 | 217.18 ± 70.20 | 0.58 | | 0.27 |
| t4-OH-Pro | 1.36 ± 6.01 | 10.58 ± 3.56 | 14.34 ± 6.40 | 10.83 ± 3.77 | 0.58 | | 0.27 |
| Ser | 104.54 ± 27.01 | 109.41 ± 27.44 | 98.89 ± 27.98 | 91.64 ± 20.65 | 0.58 | | 0.28 |
| Hexose | 6899.18 ± 2037.09 | 6507.64 ± 1109.62 | 8831.05 ± 2849.65 | 7009.23 ± 1582.43 | 0.58 | | 0.33 |
| Gln | 689.18 ± 107.76 | 633.82 ± 103.29 | 681.95± 110.96 | 611.82 ± 120.19 | 0.58 | | 0.34 |
| Ala | 305.73 ± 108.53 | 308.36 ± 90.51 | 354.73 ± 115.16 | 321.65 ± 96.29 | 0.58 | | 0.38 |
| Arg | 61.46 ± 14.79 | 71.20 ± 18.23 | 59.04 ± 14.54 | 70.60 ± 17.31 | 0.58 | | 0.40 |
| Asp | 4.71 ± 1.55 | 5.96 ± 1.57 | 4.86 ± 1.97 | 4.98 ± 1.80 | 0.59 | | 0.48 |
| Orn | 78.49 ± 24.88 | 82.77 ± 28.57 | 91.70 ± 33.28 | 80.80 ± 19.67 | 0.59 | | 0.48 |
| Asn | 38.43 ± 10.13 | 39.83 ± 10.18 | 42.64 ± 18.74 | 37.58 ± 8.61 | 0.95 | | 0.92 |
| Glu | 68.56 ± 37.02 | 104.51 ± 58.65 | 81.17 ± 34.26 | 93.11 ± 64.07 | 0.95 | | 0.95 |
| ADMA | 0.53 ± 0.31 | 0.56 ± 0.15 | 0.57 ± 90.17 | 0.53 ± 0.12 | 0.95 | | 0.95 |
| Data are presented as mean concentration [µM] ± standard deviation. For abbreviations, see Supplemental Table 1. | | | | | | | |

| **Supplemental Table 5. Repeated measures ANOVA of the metabolite concentrations grouped by the variables “T0 vs. T1” and “Diabetes mellitus type II (DM II) yes vs. no”.** | | | | | | | |
| --- | --- | --- | --- | --- | --- | --- | --- |
| **Metabolite** | **T0** | | **T1** | |  | | |
|  | **DM II yes (n=16)** | **DM II no (n=28)** | **DM II yes (n=16)** | **DM II no (n=28)** | **FDR** | **P value** | |
| SDMA | 1.14 ± 0.59 | 0.68 ± 0.23 | 0.96 ± 0.48 | 0.61 ± 0.20 | 0.57 | | 0.13 |
| Pro | 222.00 ± 61.85 | 193.43 ± 45.34 | 266.06 ± 86.24 | 219.68 ± 84.11 | 0.57 | | 0.18 |
| Orn | 84.43 ± 27.77 | 78.46 ± 26.10 | 87.41 ± 27.89 | 85.58 ± 27.84 | 0.57 | | 0.18 |
| Asp | 5.69 ± 2.10 | 5.13 ± 1.35 | 5.38 ± 1.82 | 4.66 ± 1.85 | 0.57 | | 0.21 |
| Sarcosine | 2.43 ± 13.62 | 2.20 ± 0.53 | 2.56 ± 0.66 | 2.54 ± 0.54 | 0.57 | | 0.26 |
| Cit | 35.93 ± 13.62 | 32.54 ± 9.84 | 38.84 ± 16.64 | 36.64 ± 10.19 | 0.57 | | 0.27 |
| Ala | 343.88 ± 114.62 | 286.00 ± 83.48 | 360.34 ±118.23 | 325.54 ± 104.97 | 0.57 | | 0.28 |
| Ser | 111.01 ± 33.92 | 104.680 ± 22.40 | 91.53 ± 29.71 | 97.40 ± 21.06 | 0.57 | | 0.31 |
| Hexose | 8048.25 ± 1966.22 | 5934.93 ± 674.50 | 10383.44 ± 2193.68 | 6512.54 ± 1317.54 | 0.57 | | 0.33 |
| Gly | 291.06 ± 117.50 | 248.360 ± 93.45 | 226.88 ± 104.08 | 225.68 ± 71.81 | 0.57 | | 0.35 |
| t4-OH-Pro | 13.57 ± 6.10 | 11.06 ± 4.22 | 12.45 ± 3.76 | 12.66 ± 6.27 | 0.78 | | 0.58 |
| Gln | 710.38 ± 102.46 | 633.57 ± 102.79 | 669.81 ± 141.72 | 633.79 ± 103.30 | 0.78 | | 0.59 |
| Arg | 64.21 ± 17.16 | 67.54 ± 17.27 | 59.22 ± 16.10 | 68.02 ± 16.41 | 0.86 | | 0.70 |
| ADMA | 0.60 ± 0.13 | 0.51 ± 0.13 | 0.61 ± 0.19 | 0.51 ± 0.11 | 0.89 | | 0.77 |
| Glu | 87.33 ± 61.80 | 86.08 ± 45.88 | 105.47 ± 55.56 | 76.67 ± 45.47 | 0.95 | | 0.95 |
| Asn | 43.28 ± 10.80 | 36.76 ± 8.98 | 38.81 ± 10.53 | 40.85 ± 16.48 | 0.95 | | 0.95 |
| Data are presented as mean concentration [µM] ± standard deviation. DM II, Diabetes mellitus type II. For other abbreviations, see Supplemental Table 1. | | | | | | | |

| **Supplemental Table 6. Repeated measures ANOVA of the metabolite concentrations grouped by the variables “T0 vs. T1” and “normal LV-EF yes vs. no”.** | | | | | | | |
| --- | --- | --- | --- | --- | --- | --- | --- |
| **Metabolite** | **T0** | | **T1** | |  | | |
|  | **Normal LVE-EF yes (n=34)** | **Reduced LV-EF (n=10)** | **Normal LVE-EF (n=34)** | **Reduced LV-EF (n=10)** | **FDR** | **P value** | |
| Asp | 5.68 ± 1.71 | 4.18 ± 0.85 | 5.17 ± 1.82 | 5.13 ± 1.87 | 0.06 | | **<0.01** |
| Cit | 32.20 ± 11.33 | 39.11 ± 10.30 | 35.62 ± 12.08 | 34.76 ± 12.95 | 0.40 | | **0.05** |
| Hexose | 6792.44 ± 1729.74 | 6400.70 ± 1309.73 | 7870.44 ± 2334.25 | 7649.64 ± 2909.03 | 0.40 | | 0.10 |
| Pro | 207.38 ± 56.44 | 191.70 ± 41.07 | 237.91 ± 83.24 | 230.29 ± 66.90 | 0.40 | | 0.15 |
| SDMA | 0.78 ± 0.35 | 1.09 ± 0.65 | 0.70 ± 0.32 | 0.67 ± 0.47 | 0.40 | | 0.15 |
| Sarcosine | 2.22 ± 0.57 | 2.50 ± 0.40 | 2.51 ± 0.59 | 2.44 ± 0.58 | 0.40 | | 0.16 |
| Gly | 259.41 ± 111.34 | 279.10 ± 77.17 | 225.82 ± 87.42 | 224.00 ± 78.92 | 0.40 | | 0.20 |
| Ser | 107.93 ± 26.25 | 103.75 ± 30.50 | 98.41 ± 25.01 | 100.18 ± 21.09 | 0.40 | | 0.22 |
| Ala | 327.12 ± 100.67 | 238.80 ± 58.81 | 350.15 ± 100.948 | 344.25 ± 118.32 | 0.40 | | 0.22 |
| t4-OH-Pro | 11.50 ± 5.14 | 13.56 ± 4.77 | 12.00 ± 4.37 | 11.82 ± 8.05 | 0.51 | | 0.38 |
| Orn | 83.84 ± 29.19 | 69.71 ± 11.28 | 87.03 ± 29.61 | 85.51 ± 20.74 | 0.51 | | 0.39 |
| Asn | 38.98 ± 10.45 | 39.63 ± 9.17 | 39.02 ± 8.49 | 38.70 ± 26.48 | 0.51 | | 0.40 |
| Gln | 658.09 ± 114.78 | 672.40 ± 86.14 | 648.21 ± 114.02 | 642.71 ± 141.61 | 0.51 | | 0.41 |
| Arg | 66.59 ± 18.56 | 65.46 ± 12.06 | 67.26 ± 17.54 | 69.19 ± 11.70 | 0.93 | | 0.81 |
| ADMA | 0.54 ± 0.13 | 0.56 ± 0.17 | 0.55 ± 0.16 | 0.55 ± 0.13 | 0.94 | | 0.91 |
| Glu | 94.27 ± 55.19 | 60.24 ± 27.46 | 86.72 ± 46.97 | 82.84 ± 67.68 | 0.94 | | 0.94 |
| Data are presented as mean concentration [µM] ± standard deviation. Normal LV-EF was defined as > 55%. LV-EF, left ventricular ejection fraction. For other abbreviations, see Supplemental Table 1. | | | | | | | |

| **Supplemental Table 7. Repeated measures ANOVA of the metabolite concentrations grouped by the variables “T0 vs. T1” and “Creatinine > or <1.2mg/dl”.** | | | | | | | |
| --- | --- | --- | --- | --- | --- | --- | --- |
| **Metabolite** | **T0** | | **T1** | |  | | |
|  | **Creatinine > 1.2mg/dl (n=14)** | **Creatinine < 1.2mg/dl (n=30)** | **Creatinine > 1.2mg/dl (n=14)** | **Creatinine < 1.2mg/dl (n=30)** | **FDR** | **P value** | |
| Pro | 197.50 ± 51.88 | 206.77 ± 53.49 | 227.21 ± 64.24 | 240.90 ± 85.84 | 0.24 | | **0.01** |
| Hexose | 7171.29 ± 1853.95 | 6485.07 ± 1639.38 | 8773.21 ± 2512.31 | 7522.03 ± 2358.98 | 0.70 | | 0.12 |
| Sarcosine | 2.44 ± 0.55 | 2.21 ± 0.54 | 2.65 ± 0.68 | 2.50 ± 0.54 | 0.70 | | 0.13 |
| Ala | 279.79 ± 111.37 | 319.77 ± 97.67 | 328.39 ± 135.33 | 342.77 ± 91.18 | 0.74 | | 0.20 |
| SDMA | 1.31 ± 0.54 | 0.63 ± 0.45 | 1.07 ± 0.46 | 0.58 ± 0.16 | 0.74 | | 0.26 |
| Ser | 102.88 ± 25.98 | 108.89 ± 26.97 | 84.45 ± 26.09 | 100.31 ± 22.55 | 0.74 | | 0.31 |
| Asp | 5.54 ± 2.18 | 5.24 ± 1.67 | 5.56 ± 2.02 | 4.62 ± 1.75 | 0.74 | | 0.34 |
| Gly | 277.64 ± 95.30 | 257.47 ± 102.62 | 207.43 ± 65.14 | 234.83 ± 92.03 | 0.74 | | 0.37 |
| Orn | 97.09 ± 28.00 | 72.95 ± 27.20 | 93.83 ± 24.45 | 82.71 ± 28.66 | 0.83 | | 0.48 |
| Cit | 44.39 ± 10.45 | 28.81 ± 11.41 | 42.91 ± 12.60 | 34.61 ± 11.77 | 0.83 | | 0.52 |
| Gln | 720.07 ± 108.56 | 634.17 ±107.50 | 652.79 ± 121.78 | 644.13 ± 120.34 | 0.94 | | 0.71 |
| Glu | 91.07 ± 43.21 | 84.42 ± 51.51 | 102.84 ± 59.16 | 79.82 ± 46.05 | 0.94 | | 0.79 |
| Arg | 66.57 ± 18.34 | 66.22 ± 16.98 | 57.41 ± 10.49 | 68.28 ± 18.30 | 0.94 | | 0.81 |
| t4-OH-Pro | 15.10 ± 5.71 | 10.51 ± 5.37 | 12.12 ± 3.70 | 12.80 ± 6.20 | 0.94 | | 0.82 |
| Asn | 40.86 ± 9.31 | 38.32 ± 10.05 | 39.16 ± 10.37 | 40.55 ± 16.45 | 0.94 | | 0.89 |
| ADMA | 0.65 ± 0.15 | 0.50 ± 0.14 | 0.63 ± 0.21 | 0.51 ± 0.09 | 0.94 | | 0.94 |
| Data are presented as mean concentration [µM] ± standard deviation For abbreviations, see Supplemental Table 1. | | | | | | | |

| **Supplemental Table 8. Repeated measures ANOVA of the metabolite concentrations grouped by the variables “T0 vs. T1” and “NTpro-BNP > or < 1038ng/l”.** | | | | | | | |
| --- | --- | --- | --- | --- | --- | --- | --- |
| **Metabolite** | **T0** | | **T1** | |  | | |
|  | **NTpro-BNP > 1038ng/l (n=21)** | **NTpro-BNP < 1038ng/l (n=23)** | **NTpro-BNP > 1038ng/l (n=21)** | **NTpro-BNP < 1038ng/l (n=23)** | **FDR** | **P value** | |
| Ser | 104.75 ± 27.41 | 109.17 ± 26.80 | 94.40 ± 24.19 | 96.06 ± 25.43 | 0.42 | | 0.07 |
| Pro | 203.05 ± 52.22 | 204.52 ± 54.44 | 239.71 ± 78.14 | 233.65 ± 81.30 | 0.42 | | 0.08 |
| Sarcosine | 2.27 ± 0.52 | 2.30 ± 0.56 | 2.51 ± 0.61 | 2.59 ± 0.57 | 0.42 | | 0.09 |
| SDMA | 0.98 ± 0.53 | 0.73 ± 0.34 | 0.87 ± 0.41 | 0.62 ± 0.28 | 0.42 | | 0.16 |
| Ala | 301.76 ± 93.67 | 311.87 ± 103.18 | 332.16 ± 121.73 | 343.70 ± 92.11 | 0.42 | | 0.17 |
| Gly | 281.29 ± 111.66 | 248.00 ± 93.54 | 234.90 ± 98.44 | 218.09 ± 70.87 | 0.42 | | 0.17 |
| Hexose | 6286.86 ± 1444.17 | 7083.74 ± 1725.13 | 7786.33 ± 2293.55 | 8042.30 ± 2629.78 | 0.42 | | 0.18 |
| Cit | 36.43 ± 10.30 | 31.34 ± 12.00 | 41.51 ± 12.39 | 33.36 ± 11.58 | 0.43 | | 0.22 |
| Asp | 5.35 ± 1.89 | 5.33 ± 1.44 | 5.19 ± 1.86 | 4.67 ± 1.88 | 0.59 | | 0.37 |
| Orn | 84.80 ± 32.04 | 76.83 ± 22.40 | 95.10 ± 33.44 | 78.17 ± 18.13 | 0.59 | | 0.37 |
| t4-OH-Pro | 12.77 ± 5.11 | 11.24 ± 4.94 | 13.03 ± 5.69 | 12.18 ± 5.36 | 0.63 | | 0.45 |
| Gln | 661.71 ± 96.83 | 661.30 ± 116.72 | 632.90 ± 126.02 | 659.65 ± 114.48 | 0.63 | | 0.49 |
| Arg | 61.83 ± 16.17 | 70.44 ± 16.92 | 41.11 ± 18.90 | 70.33 ± 17.45 | 0.63 | | 0.51 |
| Asn | 38.27 ± 6.98 | 39.92 ± 12.08 | 41.11 ± 18.90 | 39.20 ± 9.56 | 0.70 | | 0.61 |
| Glu | 95.55 ± 60.91 | 78.31 ± 41.61 | 95.93 ± 53.15 | 79.12 ± 49.02 | 0.85 | | 0.80 |
| ADMA | 0.55 ± 0.12 | 0.54 ± 0.15 | 0.57 ± 0.17 | 0.53 ± 0.12 | 0.90 | | 0.90 |
| Data are presented as mean concentration [µM] ± standard deviation. NTpro-BNP, N-terminus pro-B type natriuretic peptide. For other abbreviations, see Supplemental Table 1. | | | | | | | |

| **Supplemental Table 9. Association of different independent parameters others than “T0 vs. T1” with metabolite concentrations in the linear mixed model regression analysis.** | | | | | | | |
| --- | --- | --- | --- | --- | --- | --- | --- |
| **Metabolite** | **Variable** | **Beta** | **SE** | **T value** | **CI (2.5%)** | **CI (97.5%)** | **P value** |
| ADMA | Creatinine | 0.28 | 0.10 | 2.73 | 0.09 | 0.47 | **0.01** |
| Ala | Diabetes mellitus II | 0.34 | 0.15 | 2.26 | 0.07 | 0.62 | **0.03** |
| Ala | LV-EF | -0.30 | 0.15 | -2.04 | -0.57 | -0.03 | **0.049** |
| Arg | NTpro-BNP | -0.22 | 0.09 | -2.33 | -0.28 | -0.09 | **0.025** |
| Asp | LV-EF | -0.40 | 0.15 | -2.65 | -0.44 | -0.38 | **0.012** |
| Cit | Creatinine | 0.43 | 0.14 | 3.12 | 0.18 | 0.69 | **0.004** |
| Gly | Gender | -0.34 | 0.15 | -2.23 | -0.61 | -0.06 | **0.032** |
| Hexose | Diabetes mellitus II | 0.55 | 0.09 | 6.05 | 0.39 | 0.72 | **<0.001** |
| Orn | Creatinine | 0.29 | 0.14 | 2.10 | 0.04 | 0.54 | **0.042** |
| Pro | Diabetes mellitus II | 0.30 | 0.14 | 2.14 | 0.05 | 0.56 | **0.039** |
| SDMA | Creatinine | 0.67 | 0.14 | 4.75 | 0.42 | 0.93 | **<0.001** |
| SDMA | Gender | -0.29 | 0.13 | -2.26 | -0.53 | -0.06 | **0.03** |
| SDMA | NTpro-BNP | 0.26 | 0.12 | 2.14 | 0.04 | 0.49 | **0.039** |
| Metabolites and their association to independent variables, appearing in alphabetical order. Results are shown only when p < 0.05. CI, confidence interval; SE, standard error. For definitions and abbreviations of variables, see legends of supplemental tables 1-7. | | | | | | | |
